# Innate immune receptors for cross-presentation: The expanding role of NLRs

**DOI:** 10.1016/j.molimm.2017.11.028

**Published:** 2019-09

**Authors:** Daniele Corridoni, Alison Simmons

**Affiliations:** a MRC Human Immunology Unit, Weatherall Institute of Molecular Medicine, John Radcliffe Hospital, University of Oxford, Oxford, OX3 9DS, UK; b Translational Gastroenterology Unit, John Radcliffe Hospital, University of Oxford, Oxford OX3 9DU, UK

**Keywords:** PRRs, NLRs, Cross-presentation, MHC class I, CD8+ T cells

## Abstract

A critical role of pattern recognition receptors (PRRs) is to influence adaptive immune responses by regulating antigen presentation. Engagement of PRRs in dendritic cells (DCs) increases MHC class I antigen presentation and CD8^+^ T-cell activation by cross-presented peptides but the molecular mechanisms underlying these effects are not completely understood. Studies looking at the role of PRRs in cross-presentation have been largely limited to TLRs but the role of other PRRs such as cytosolic nucleotide-binding oligomerization domain-like (NOD-like) receptors remains particularly enigmatic. Here we discuss recent evidence of the role of PRRs on cross-presentation and consider how cytosolic NLR-associated pathways, such as NOD2, may integrate these signals resulting in effective adaptive CD8^+^ T cells responses.

## Highlights

• PRRs temporally control cross-presentation during acute vs. chronic pathogen handling.
• NLRs signal in close proximity to phagosomal and endosomal membranes.
• Current status of NLR-dependent regulation of MHC class antigen presentation.

## 1 Introduction

Major histocompatibility complex (MHC) class I (MHC-I) molecules present internalized exogenous antigens in a process called cross-presentation (Burgdorf et al., 2008). It is now well recognized that cross-presentation is a critical mechanism for establishing tolerance to self-antigen but also for priming adaptive immune responses against microbial pathogens and tumors (Joffre et al., 2012). Different pathways and subcellular locations have been proposed to regulate cross-presentation, including the vacuolar and phagocytic (cytosolic) pathways. The vacuolar pathway involves the degradation of antigens by endosomal or phagosomal proteases, in particular cathepsin S, and the resultant peptides are loaded onto MHC class I molecules independent of degradation by the immunoproteasome (Shen et al., 2004). In the case of the phagocytic pathway, internalized antigens from endosomes or phagosomes are exported to the cytosol where they are degraded by the immunoproteasome. This leads to the generation of peptides that are then transported back into the phagosome via TAP and loaded onto MHC-I (Burgdorf et al., 2008, Houde et al., 2003). Alternatively, peptides are transported via TAP into the endoplasmic reticulum (ER), for loading onto ER-resident heavy chain-B2 m complexes (Kovacsovics-Bankowski and Rock, 1995). Although these results suggest that the immunoproteasome is necessary for cross-presentation, there is no evidence suggesting that the immunoproteasome is the exclusive cytosolic protease acting in the phagocytic pathway.

During cross-presentation the establishment of CD8^+^ T-cell-mediated responses is ultimately dictated by professional antigen presenting cells (APCs), such as dendritic cells (DCs) whose function is to acquire exogenous antigens and direct the formation of a complex between the MHC-I peptide and a cognate TCR to direct the activation and proliferation of antigen-specific T cells (Joffre et al., 2012). A second signal from DCs is necessary to determine successful activation of naïve T cells and it is controlled by innate immune sensing of microbial components through the pattern recognition receptors (PRRs) (Akira et al., 2006). Engagement of PRRs induces a significant change in the phenotype and maturation of DCs characterized by enhanced expression of co-stimulatory molecules and increased secretion of cytokines necessary for activation of naïve T cells (Reis e Sousa, 2006). Signals derived from PRRs determine whether cross-presentation leads to activation or inactivation (cross-tolerance) of T cells depending on whether or not DCs are exposed to PRRs ligands and on the timing of this exposure (Heath and Carbone, 2001). Physiologically, this contributes to minimize the risk of generating a response to self antigens, to maximize T-cell priming against exogenous antigens during the transient phase of DC maturation, and to temporally control MHC class I antigen presentation and prevent excessive priming during chronic phases of pathogen handling.

Because PRR signaling enhances co-stimulatory signals, antigen capture can delay phagosome maturation to promote efficient antigen presentation in DCs, all of which activate T cells, the direct effect of PRRs engagement on cross-presentation has been difficult to determine. However, a number of recent studies have demonstrated that PRR signaling may impact on multiple processes associated with cross-presentation of peptides. Whether distinctions occur in terms of the mechanism by which different classes of PRRs contribute to cross-presentation is not completely understood. In this regard, the effect of cytosolic NLRs remains particularly enigmatic.

## 2 Current status: signals from PRRs

### 2.1 TLRs

Different mechanisms have been described to explain the increased cross-presentation observed during the initial phase of TLR sensing in immature DCs including a recent one from Blander and colleagues who showed that the recruitment of MHC class I molecules to phagosomes is enhanced by TLR4 stimulation (Nair-Gupta et al., 2014). In their work, MHC-I molecules are showed to be stored in phagosomes from endosomal recycling compartments (ERC) containing the small GTPase Rab11a and the t-SNARE proteins VAMP3 and VAMP8. Upon LPS stimulation, TLR4 engagement leads to IKK2-dependent phosphorylation of phagosome-associated SNAP-23 (synaptosome-associated protein of 23 kDa) promoting fusion between ERCs and phagosomes. MyD88-dependent TLR signals might be compartmentalized impacting only phagosomes carrying TLR ligands and leading to phosphorylation of SNAP23 specifically on those phagosomes. Phosphorylation of SNAP23 by IKK2 may thus serve as a flag on phagosomal membranes by facing the cytosol and serving as the docking site for outgoing traffic from the ERC. This mechanism serves to deliver to phagosomes enough numbers of MHC-I molecules once TLRs signal microbial components to increase need for cross-presentation (Nair-Gupta et al., 2014, Blander, 2016).

The efficiency of MHC-I loading is also enhanced by TLRs through promotion of the NADPH oxidase NOX2 activity in DCs (Vulcano et al., 2004). NOX2 is recruited to immature DC phagosomes, causing active and sustained phagosome alkalinization by consuming protons for di-oxygen production by dismutation. In line with this, NOX2-defective DCs, displayed increased phagosome and early endosome acidification and, as a consequence, caused a defect in cross-presentation (Jancic et al., 2007, Mantegazza et al., 2008, Savina et al., 2006, Trombetta et al., 2003). This suggests that TLR-dependent recruitment of NOX2 limits acidification and degradation in phagosomes leading to decreased peptide degradation to favour antigen cross-presentation.

Another critical step in antigen cross-presentation regulated by engagement of TLR signaling is the export of internalized antigens to the cytosol (Gil-Torregrosa et al., 2004). Following a short stimulation of TLR4 by LPS in DCs (0–3 h), antigen translocation from the phagosome to the cytosol is increased. However, the mechanisms for this increase remain unclear. During the intermediate phase of DC maturation after several hours of TLR engagement (3–16 h), the efficacy of cross-presentation is still increased *in vitro* and *in vivo* (Gil-Torregrosa et al., 2004, Alloatti et al., 2015). During this phase, DCs exhibit increased endocytosis, proteasomal and TAP activity (Alloatti et al., 2016). In addition, enhanced cross-presentation in intermediate DC maturation results from delayed phagosomal degradation and decreased recruitment of lysosomal proteases to phagosomes leading to decreased acidification. Peri-nuclear clustering of lysosomes, a process mediated by the GTPase RAB34 (Kasmapour et al., 2012, Wang and Hong, 2002), together with reduced displacement of phagosomes along microtubules prevents their fusion, with the end effect of increasing the efficiency of antigen cross-presentation (Alloatti et al., 2015).

DCs that have been exposed to TLR agonists for an extended period of 24–40 h have markedly reduced efficiency of cross-presentation (Gil-Torregrosa et al., 2004). This is likely due to decreased antigen uptake or antigen export to the cytosol (Weck et al., 2007, Wilson et al., 2006). Besides this, decreased cross-presentation observed at late time points could be also due to LPS-mediated upregulation of the transcription factor TFEB, which controls the lysosomal biogenesis pathway. In a recent study Cresswell et al. showed that activation of TFEB, followed by its translocation to the nucleus, downregulated MHC class I–restricted antigen cross-presentation and cross-priming of naive CD8^+^ T cells (Samie and Cresswell, 2015). In line with this, overexpression of TFEB induced phagosomal acidification and increased expression of lysosomal enzymes in immature DCs leads to decreased cross-presentation (Samie and Cresswell, 2015).

Overall, evidence indicates there is temporal control of MHC-I antigen presentation with initial TLR engagement promoting its efficiency during the early or acute phase of microbial exposure but prolonged stimulation leading to mechanisms to prevent excessive priming during chronic phases of pathogen handling.

### 2.2 NOD-like receptors (NLRs)

The NLRs are a group of conserved intracellular PRRs that play a pivotal role in innate immunity. The NLR family includes 22 identified protein members in humans (Chen et al., 2009). The structural features of NLRs are characterized by a central nucleotide-binding oligomerization (NOD) domain, which mediates the self-oligomerization occurring during activation, a variable N-terminal protein–protein interaction domain, defined by the caspase recruitment domain (CARD), and a C-terminal leucine-rich repeat (LRR) that detects pathogen-associated molecular patterns (PAMPs). Based on the variation in their N-terminal domain, the NLR family can be further subdivided into five families: NLRA (CIITA), NLRB (NAIPs), NLRC (NOD1, NOD2, and NLRC3-5), NLRP (NLRP 1–14) and NLRX (Chen et al., 2009, Corridoni et al., 2014b). The critical function of NLRs is to sense microbial products in the host cytosol in contrast to TLRs, which recognize microbial ligands at the cell surface or within endosomes. This provides an extra layer of microbial surveillance that is often associated with pathogen invasion (Caruso et al., 2014).

**Fig. 1:**
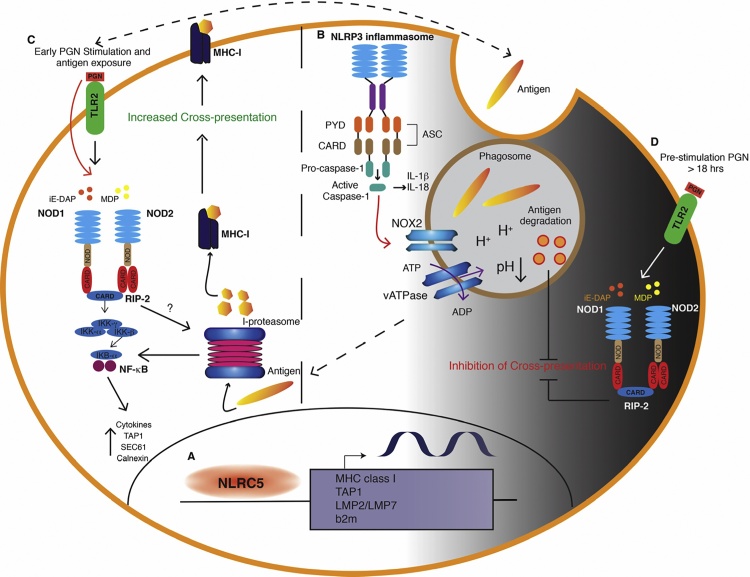
NLR-dependent regulation of MHC class I antigen presentation. (A) NLRC5 induces chromatin remodeling and recruitment of transcription factors, leading to the transactivation of MHC class I, and other related genes in the MHC class I antigen-presentation pathway, including *LMP2, LMP7*, *TAP1*, and β2-microglobulin. (B) Activation of the NLRP3 inflammasome and its effector caspase-1 acts on the NADPH oxidase NOX2 to induce phagosome acidification in response to microbial infection. This process negatively impacts cross-presentation. (C) NOD1 and NOD2 activation by peptidoglycan (PGN) significantly augments DC-mediated cross-presentation via upregulation of intracellular components, such as TAP, SEC61, and calnexin, for MHC class I dependent antigen presentation and co-stimulatory molecules expression. In addition, early activation of NOD2 signaling alone or in combination with TLR2 may be required for stabilisation of the immunoproteasome and promote cross-presentation during an early phase of pathogen handling. (D) PGN pre-treatment, prior to antigen encounter, leads to progressive inhibition of cross-presentation over time, with almost complete inhibition after 18 h.

Recent studies revealed that the NOD-like receptor (NLR) caspase recruitment domain–containing protein 5 (NLRC5) plays a key role in the transcriptional regulation of MHC class I (Meissner et al., 2010). NLRC5 does not directly bind the DNA, but it is recruited by the enhanceosome, a DNA-binding complex assembling on the SXY module. Similar to CIITA, another member of the NLR family that acts as a transcriptional regulator of MHC class II genes, NLRC5 induces chromatin remodeling and recruitment of transcription factors, leading to the transactivation of MHC class I and other related genes in the MHC class I antigen-presentation pathway, including *LMP2*/*LMP7*, *TAP1*, and β2-microglobulin (Downs et al., 2016, Ludigs et al., 2015) (Fig. 1A). A recent study from Guarda and colleagues found that NLRC5 largely contributes to *H2-K* transcription in DCs following exposure to inflammatory stimuli (i.e. LPS) (Rota et al., 2016). In line with this, presentation of endogenous antigens by *Nlrc5^−/−^* DCs was downregulated indicating that the NLRC5-dependent decrease in the amount of de novo synthesized MHC class I molecules affected the display of intracellular antigens. Despite this, the authors did not observe NLRC5-dependent modulation in the efficiency of T-cell priming and cross-priming by DCs (Rota et al., 2016). This suggests that, although NLRC5 affects MHCI transcription and direct antigen presentation in stimulated DCs, this may not be sufficient to significantly alter cross-presentation to CD8^+^ T cells.

NLRP3 consists of a carboxy-terminal LRR domain, a central NOD domain, and an amino-terminal PYD, mainly interacting with apoptosis-associated speck-like protein containing a CARD (ASC) (Schroder and Tschopp, 2010). NLRP3 participates in inflammasome formation through the recruitment of ASC, subsequent activation of caspase-1, and secretion of IL-1β and IL-18 (Corridoni et al., 2014a, Corridoni et al., 2014b, Schroder and Tschopp, 2010). Activation of the NLRP3 inflammasome and its effector caspase-1 can impact phagosome function by causing rapid and locally restricted modification of the proteins associated to the organelle, which results in induction of the microbicidal activity (Sokolovska et al., 2013). Specifically, phagosome-associated caspase-1 acts on the NADPH oxidase NOX2, and by controlling its activity it modifies the pH of the vacuole (Fig. 1B). In fact, NLRP3-deficient and caspase-1-deficient cells fail to induce phagosome acidification in response to microbial infection (Sokolovska et al., 2013). As discussed above, cross-presentation of phagocytosed antigens to CD8^+^ T cells is thought to occur primarily from a nonacidified phagosome. Therefore, activation of the inflammasome and its effector caspase-1 has been shown to negatively impact cross-presentation and this might occur primarily by controlling the pH of phgosomes, which accelerates degradation of antigens.

Despite these few examples, it is not clearly elucidated how cross-presentation is affected by NLR activity. Given that these receptors signal at sites that are potentially in close proximity to phagosomal and endosomal membranes containing high levels of bacterial components, they may play a critical role in interacting with the antigen presentation machinery.

### 2.3 NOD1 and NOD2

NOD1 and NOD2 share the same structure with exception of the amino-terminal domain, composed by a single CARD for NOD1, compared to the tandem CARDs contained within the NOD2 domain. NOD1 and NOD2 senses conserved fragments of peptidoglycan (PGN): NOD1 can be activated by γ-d-glutamyl-*meso*-diaminopimelic acid (iE-DAP), present in the cell wall of many Gram^−^ bacteria and some Gram^+^ bacteria; NOD2 can be activated by muramyl-dipeptide (MDP) widely distributed among both Gram^+^ and Gram^−^ bacteria. Activation of NOD1/NOD2 by PGN leads to intracellular signaling pathways that drive pro-inflammatory and antimicrobial responses (Caruso et al., 2014). NOD1/NOD2 activation by PGN leads to self-oligomerization and recruitment of receptor-interacting serine/threonine-protein kinase 2 (RIPK2), through homotypic CARD–CARD interactions (Fig1C). RIPK2 induces phosphorylation and ubiquitination of IκB kinase-γ, which leads to recruitment of the serine/threonine kinase TAK-1, and activation of the IKK complex and MAPK pathway (Abbott et al., 2007, Hasegawa et al., 2008, Ogura et al., 2001).

In addition to activating the NF-κB and MAPK signaling pathways, NOD1 and NOD2 signaling influences adaptive immune responses. NOD2 regulates Th17 cell responses and in mice, stimulation of either NOD1 or NOD2 induces antigen-specific immune response associated to Th2 polarization (Fritz et al., 2007, Magalhaes et al., 2008, Corridoni et al., 2017, Brain et al., 2013). In addition, co-stimulation of NOD1 and NOD2 with TLR agonists, promotes the priming of Th1, Th2 and Th17 cell immune responses. Although several studies have demonstrated a clear role for NOD1 and NOD2 signaling in regulating adaptive immune responses, it is not clear how bacterial detection by these NLRs is mechanistically linked to its role in antigen presentation. NOD2 induces autophagy in human DCs influencing bacterial killing and enhancing MHC class II presentation of bacterial antigens (Cooney et al., 2010, Travassos et al., 2010). In fact, NOD2 stimulation leads to MHC class II localization with microtubule-associated protein 1A/1B/light chain-3 (LC3), autophagy dependent upregulation of surface MHC class II and generation of antigen-specific CD4^+^ T cell responses. These studies were particularly relevant because of the known association of NOD2 and autophagy with Crohn’s disease (Cooney et al., 2010).

NOD1 and NOD2 have been previously demonstrated to modulate cross-presentation. Asano and colleagues showed that NOD1 and NOD2 activation by PGN significantly augments CD8a^+^ DC-mediated cross-presentation via upregulation of intracellular components, such as TAP, SEC61, and calnexin, for MHC class I dependent antigen presentation and co-stimulatory molecules expression. As a result, enhanced antigen-specific CD8^+^ T cells were induced on NOD triggering (Fig. 1C). In that study, the authors suggest that NOD/RIPK2-mediated signals might mimic the TLR4-MyD88 signals necessary to induce recruitment of TAP to the early endosomes, an essential step for cross-presentation of soluble antigens (Asano et al., 2010).

In another study, Cresswell and colleagues found that pre-treatment of DCs with pure PGN or both synthetic ligands for NOD1 and NOD2 impaired cross-presentation of HSV antigens and vaccinia virus-expressed OVA (VV-OVA) (Wagner and Cresswell, 2012). In this study, PGN pre-treatment led to progressive inhibition of cross-presentation over time, with decreased cross-presentation after 12 h and almost complete inhibition after 18 h (Wagner and Cresswell, 2012). The study from Cresswell and colleagues examined how maturation of DCs prior to antigen encounter modulates cross-presentation, which clearly shows that NOD1 and NOD2 activation abrogated cross-presentation of HSV antigens even in the presence of an LPS signal that would otherwise enhance it. Thus, as described for TLR signals, NOD receptors effect on cross-presentation can be subdivided temporally into three main phases: early, intermediate and late. During the early and intermediate phase of DC maturation, activation of NOD1 and NOD2 signaling alone or together with other PRRs enhances cross-presentation while during the late phase DC maturation directed by PRR stimulation lead to decreased cross-presentation (Fig. 1D).

It has been largely demonstrated that co-stimulatory signals to TLR and NLR have synergistic effects. Under physiological conditions, DCs often meet a combination of TLR and NLR ligands (Girardin et al., 2003, Netea et al., 2005). The cross-talk between NOD2 and TLR2 has been extensively described as both receptors recognize adjacent components of PGN expressed on bacterial cell walls allowing these receptors to amplify the response not only to a single pathogen but also to a single component of a pathogen. Whether distinctions occur in terms of the molecular mechanisms by which different classes of TLRs and NLRs connect to MHC class I antigen presentation machinery is not known. Our group has used quantitative phosphoproteomic analysis to define NOD2 signaling in primary human DCs and to compare NOD2 stimulation with TLR2 stimulation alone or in combination by stimulating DCs with either MDP and PAM_3_CSK_4_ or a combination of both ligands. We found that early activation of NOD2 signaling alone or in combination with TLR2 may affect the function of immunoproteasome subunits and promote cross-presentation to CD8^+^ T cell (manuscript in preparation). Thus, this signaling pathway may mechanistically explain how engagement of NOD2 upon PGN stimulation promotes MHC class I antigen presentation during an early phase of pathogen handling (Fig. 1C). In addition, given that long pre-treament of DCs with PGN leads to impaired cross-presentation (Wagner and Cresswell 2012), this signaling pathway could also be a means to temporally control MHC class I antigen presentation and prevent excessive priming during chronic phases of pathogen handling. In fact, as the synergistic crosstalk between NOD2 and other PRRs, including TLR2, risks overactivation of the immune system with potentially deleterious consequences, it is unsurprising that suppressive mechanisms may have also evolved in the context of MHC class I antigen presentation. This might be highly relevant to the pathogenesis of chronic inflammatory disorders such as Crohn’s disease, in which loss of function associated polymorphisms in the NOD2 gene are associated with increased susceptibility to disease.

### 2.4 Future perspectives

Bacterial cell wall components represent a major source of adjuvants given their strong immunostimulatory effects. MDP was identified in 1974 as the minimal active mycobacterial component responsible for the potent activity of complete Freund’s adjuvant (CFA) (Adam et al., 1974, Ellouz et al., 1974). More than 30 years later, MDP was revealed to activate NOD2 to optimally mount innate and humoral immune responses. The adjuvant activity of MDP and TLR ligands is well known to enhance vaccine responses and our increasing understanding of the molecular mechanisms associated with these effects may lead to potential new targets for adjuvant development (Maisonneuve et al., 2014). There is a growing need for new, safe, and nontoxic adjuvants that are more effective in inducing long-lasting protective responses to vaccination and CD8 cellular immunity.

Polymorphisms in NOD2 remain the strongest known genetic risk factors in the development of Crohn’s disease. Three common NOD2 variants (R702W, G908R, and L1007insC) and multiple minor variants in the C-terminal LRR region and HD2 are linked to the development of Crohn’s disease (Caruso et al., 2014, Corridoni et al., 2014a, Corridoni et al., 2014b). The precise mechanism by which Crohn’s associated NOD2 variants might initiate and perpetuate chronic inflammatory diseases has not been firmly established. Increasing evidence suggests that CD8^+^ T cells may play an earlier role in inflammatory bowel disease development than the CD4^+^ T cells, which are more traditionally associated with disease pathogenesis (Lee et al., 2011, Nancey et al., 2006, Steinhoff et al., 1999, Westendorf et al., 2006). Whether dysregulated NOD2 signaling occurring in the presence of NOD2 polymorphisms may also affect CD8^+^ T cell responses by aberrant priming via the cross-presentation pathway remains to be explored. Therefore, answering these questions in the near future may provide the rationale for novel pharmacologic strategies aimed to modulate CD8^+^ T cells responses.

## Conflicts of interest

There are no financial conflicts of interest to declare.
